# Dietary Behaviors and Mental Health Disorders Among Private University Students in Dhaka North City Corporation, Bangladesh: A Cross‐Sectional Study

**DOI:** 10.1002/puh2.70283

**Published:** 2026-05-28

**Authors:** Mst. Mahfuza Akter, Taslima Khatun, Md. Nazmus Sakib, Sheikh Mohammed Shariful Islam

**Affiliations:** ^1^ Department of Community Nutrition Bangladesh University of Health Sciences Dhaka Bangladesh; ^2^ Department of Disaster Management & Resilience Bangladesh University of Professionals Dhaka Bangladesh; ^3^ Department of Nutritional Science Texas Tech University Lubbock Texas USA

**Keywords:** Bangladesh, eating habits, mental health, private university students

## Abstract

**Background:**

University students around the world are experiencing rising levels of psychological issues, particularly depression, anxiety, and stress. Poor dietary habits may exacerbate these conditions, yet limited research has examined this relationship among private university students in Bangladesh.

**Aims:**

This study aims to examine the relationship between dietary behaviors and mental health outcomes among students at private universities in Bangladesh.

**Methods:**

A cross‐sectional survey was conducted between December 2023 and May 2024 among 384 private university students selected using convenience sampling. Data were collected using a structured questionnaire, and multivariate logistic regression analyses were performed to identify factors associated with depression, anxiety, and stress.

**Results:**

Among participants, 59.4% reported depression, 63.3% anxiety, and 45.3% stress, ranging from mild to extremely severe. Associations were observed between mental health outcomes and gender, academic level, meal patterns, and junk food consumption. Females had lower odds of anxiety (odds ratio [OR] = 0.41) and stress (OR = 0.48) compared to males. Undergraduate students were less likely to report anxiety than postgraduate students (OR = 0.17). Regular breakfast and lunch intake was associated with lower odds of stress (OR = 0.52), depression (OR = 0.50), and anxiety (OR = 0.44). Preference for rice and fish was associated with lower depression odds (OR = 0.26) relative to fast food, sweets, and processed products. Alcohol consumption was associated with higher stress odds (OR = 2.65).

**Conclusions:**

Unhealthy eating behaviors were associated with higher levels of depression, anxiety, and stress, whereas regular meals and traditional dietary choices were associated with lower odds of mental health problems. These findings highlight the need for integrated nutritional and mental health interventions to support university students’ psychological well‐being.

## 1 Introduction

Although health is critically important from early life, mental health has historically received less attention than physical health [1]. Although physical health issues often have clear definitions and observable symptoms, mental health is influenced by cultural, traditional, economic, and geographic factors [2] and is a vital component of overall well‐being, commonly assessed through levels of stress, anxiety, and depression [3]. Globally, mental health among university students is an increasing concern [4, 5], with one in four young people experiencing a mental disorder annually [6], and approximately 75% of these conditions emerging before age 24 years [7]. In South Asia, the prevalence of mental health disorders among university students is notably high, with depression at 29.4%, anxiety at 42.4%, and stress at 16.4% [8]. Similarly, studies in Bangladesh indicate a substantial burden of depression, anxiety, and stress among university students [5, 9, 10].

University students are particularly vulnerable to these challenges as they navigate the critical stage of identity formation [11]. Multiple factors contribute to elevated depression, anxiety, and stress in this population, including academic pressures, social adaptation, and future careers [12, 13, 14]. For students in later years of university, these challenges are further intensified by the combined demands of psychosocial adjustments and academic and social expectations [15]. Additionally, inadequate nutrient intake and imbalanced eating patterns have emerged as global concerns, negatively affecting mental health and increasing the risk of depression, anxiety, stress, and impaired academic performance [16, 17].

Dietary habits are closely linked with mental health. Many students frequently consume fast food, sugary snacks, and processed items, which are calorie‐dense but nutrient‐poor items, leading to deficiencies in vitamins and minerals [16, 18, 19]. These poor dietary practices can influence fatigue, reduced concentration, weakened immunity, and adverse mental health outcomes [16, 20, 21]. Additionally, skipping meals is a common practice among students, which can result in nutritional imbalances and energy deficiencies that negatively impact academic performance [22].

Despite increasing attention to mental health, there remains a notable gap in research exploring the intersection of diet and mental well‐being in Bangladesh, particularly within private universities. Existing studies have largely focused on students from public institutions [23], overlooking potential differences in academic environments, lifestyles, and psychological stressors between public and private universities. To address this gap, the present study examined the association between dietary behaviors and mental health outcomes, including depression, anxiety, and stress, among students at private universities in Bangladesh.

## 2 Materials and Methods

### 2.1 Study Design

This cross‐sectional study was conducted between December 2023 and May 2024 among university students.

### 2.2 Eligibility Criteria

#### 2.2.1 Inclusion Criteria

- Being Bangladeshi by birth;
- Aged 18–29 years;
- Current undergraduate or postgraduate student of the selected university.

#### 2.2.2 Exclusion Criteria

- Incomplete or missing questionnaire responses;
- Non‐cooperative students or those who refused to participate.

### 2.3 Setting and Participants

Students were recruited from four private universities in Dhaka North City Corporation: Bangladesh University of Health Sciences (*n* = 88), ASA University (*n* = 106), European University (*n* = 96), and Prime University (*n* = 94).

The minimum sample size was calculated using the following formula:

$$n=\frac{z^{2}\times p\times \left(1-p\right)}{d^{2}}$$

where *z* = 1.96 (95% confidence level), *p* = 55.9% [24] (expected proportion of students with poor mental health), and *d* = 0.05 (margin of error). The minimum required sample was 379 participants. The total student population of the selected universities was not available, which may limit the assessment of sample representativeness.

**FIGURE 1:**
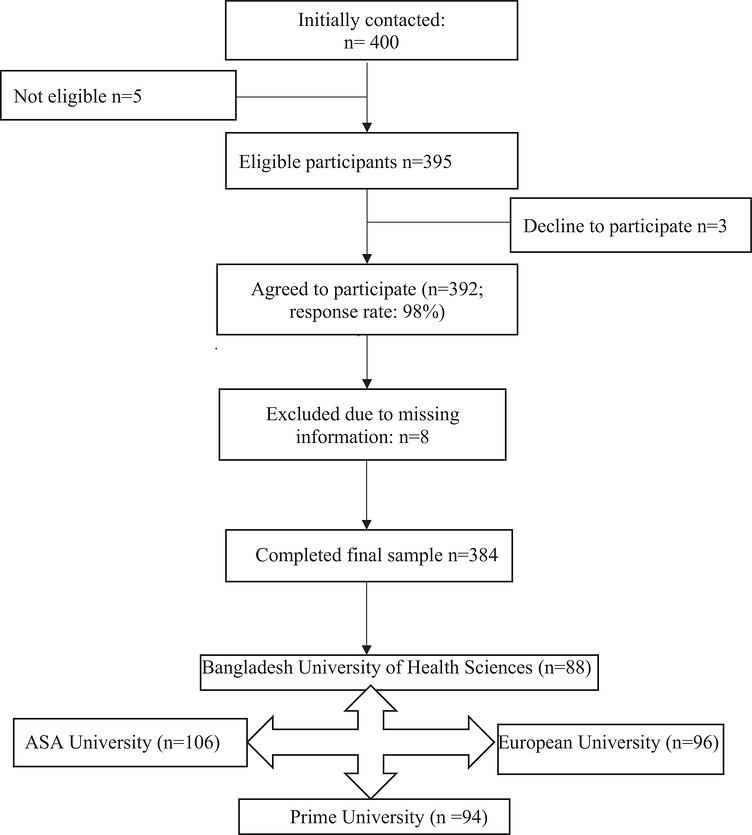
Flow chart of the sample collection.

A non‐probability convenience sampling technique was employed to recruit universities and participants. Students present on campus during the data collection period who voluntarily agreed to participate were included. Although this approach allowed efficient data collection, it may limit the generalizability of the findings. To account for potential non‐response, 400 students were initially contacted. Among them, 395 were eligible, and 392 agreed to participate (response rate: 98%). After data screening, eight questionnaires were excluded due to missing information, resulting in a final analytical sample of 384 participants (Figure 1).

## 3 Study Measures

### 3.1 Independent Variable

#### 3.1.1 Sociodemographic Characteristics

Sociodemographic information was recorded during the survey, including age, height, weight, sex (male/female), educational status (undergraduate/postgraduate), family size, members (nuclear family ≤ 5/extended family > 5), residence (with family/hostel/mess), religion (Islam/others), and family monthly income, Bangladesh Taka (BDT) (<20,000/20,000–50,000/>50,000).

#### 3.1.2 Body Mass Index (BMI)

BMI was calculated using directly measured height and weight, with the formula BMI = kg/m^2^. According to the World Health Organization (WHO) guidelines for the Asian population, BMI results were categorized into four groups: underweight (BMI < 18.5); normal weight (18.5 ≤ BMI ≤ 24.9); overweight (25 ≤ BMI ≤ 29.9); and obese (BMI ≥ 30) [25].

#### 3.1.3 Anthropometric Assessment

Height and weight measurements were conducted by trained data collectors following standardized procedures. Measurements were taken in a private classroom, and participants were asked to remove shoes, socks, and any heavy clothing or accessories. Weight was recorded using a Camry EB9062 electronic personal scale (Made in China), whereas height was measured to the nearest 0.1 cm using a non‐stretchable tape.

### 3.2 Dietary Assessment

The 19‐item questionnaire was developed to capture multiple domains of dietary behavior, including meal frequency (e.g., breakfast, lunch, and dinner), snacking habits (e.g., fried snacks, junk food, sweets, and processed items food), beverage consumption (e.g., tea, soft drinks, and energy drinks), and preferences for culturally relevant foods (e.g., shingara, puri, and tea), are commonly found in university cafeterias [19, 23, 26, 27, 28]. A pilot survey with 30 participants, recruited from a separate private university to assess clarity and usability. Participants generally found the items understandable and relevant. Only a few students reported consuming certain foods exactly 3 days/week, which informed our decision to collapse the original three‐category frequency variable (<3, 3, and >3 days/week) into two categories (<3 vs. >3 days/week) for the main study. No other modifications were necessary based on pilot feedback. The internal consistency of the questionnaire was acceptable (Cronbach's *α* = 0.72 for all 19 items). The questionnaire was administered in English because the target participants (undergraduate and graduate students) had sufficient English proficiency, which is the medium of instruction for most courses at the participating universities. Data from the pilot participants were excluded from subsequent analyses. The adapted 19‐item questionnaire, which has not been published elsewhere, has been uploaded as an English‐language Supporting Information File 1.

### 3.3 Dependent Variable

#### 3.3.1 Mental Health Disorders

Mental health disorders were measured using the depression, anxiety, and stress scale (DASS‐21). It is composed of three subscales, each containing seven items, each measuring a distinct aspect of negative affect: depression (sadness, hopelessness, and loss of interest), anxiety (nervousness, tension, and worry), and stress (difficulty relaxing, irritability, and feeling overwhelmed) [29]. Responses were recorded on a 4‐point Likert scale (0 = never, 1 = sometimes, 2 = often, and 3 = almost always). To assess severity, scores for each subscale were obtained by summing the item responses and multiplying the total by two. Severity was classified as follows: normal (depression: 0–9, anxiety: 0–7, and stress: 0–14), mild (depression: 10–13, anxiety: 8–9, and stress: 15–18), moderate (depression: 14–20, anxiety: 10–14, and stress: 19–25), severe (depression: 21–27, anxiety: 15–19, and stress: 26–33), and extremely severe (depression: 28+, anxiety: 20+, and stress: 34+).

### 3.4 Ethical Issues

Permission for data collection was granted by the respective universities’ administrations. Written informed consent was obtained from all participating students. Participants received a detailed consent form outlining the study's purpose, procedures, potential risks, confidentiality, and anonymity.

### 3.5 Data Collection Procedure

Data were collected from participants using a printed structured questionnaire administered by the data collectors. The principal investigator (PI) organized a training program to instruct the data collectors on data collection procedures, scheduling, and the participants’ eligibility. Four data collectors visited the selected universities and carried out the data collection. Each data collection session took approximately 15–20 min.

### 3.6 Statistical Analysis

Data were analyzed using R software (version 4.3.2). Descriptive statistics were used to summarize data: Continuous variables were presented as means and standard deviations (SDs) when appropriate, and categorical variables were presented as counts and percentages. The bivariate chi‐square test was applied to examine the proportional difference between eating habits and mental health outcomes. Variables with *p* values < 0.20 in the bivariate analysis were entered into the multivariate logistic regression model [30, 31]. Before running the multivariate models, all independent variables were checked for multicollinearity using the variance inflation factor (VIF), and no significant multicollinearity was detected (all VIF values < 2). Multivariate logistic regression analysis was then performed to identify factors independently associated with mental health outcomes, and odds ratios (ORs) with 95% confidence intervals (CIs) were calculated. Statistical significance was set at ≤0.05. Sensitivity analyses were conducted by re‐running the regression models after excluding selected variables and assessing the impact of potential outliers. This study adhered to the Strengthening the Reporting of Observational Studies in Epidemiology (STROBE) guidelines, and the completed STROBE checklist was provided in Supporting Information File 2.

## 4 Results

### 4.1 General Characteristics of the Study Participants

A total of 384 students participated in the study. The mean age was 21.96 years (SD = ±1.80), and mean BMI was 23.54 kg/m^2^ (SD = ±4.97). About 62% were aged 18–22 years, and 54% were male. Most participants were undergraduates (96%), identified as Muslim (88%), and came from families with five or fewer members (77%). Approximately 61% lived with their families, and 56% reported a monthly family income of 20,000–50,000 BDT. Regarding nutritional status, 54% were of normal weight, 15% underweight, 20% overweight, and 11% obese (Table 1).

**TABLE 1:** Demonstrates the demographic characteristics of the participants (*N* = 384).

Variables	*n* (%)
**Age, years (Mean ± SD)**	21.96 ± 1.80
**BMI (kg/m^2^)**	23.54 ± 4.97
**Age category**	
18–22	238 (62)
23–27	146 (38)
**Sex**	
Male	209 (54)
Female	175 (46)
**Educational status**	
Undergraduate	367 (96)
Postgraduate	17 (4)
**Religion** ^a^	
Islam	337 (88)
Others	47 (12)
**Family size, members**	
Nuclear family (≤5)	295 (77)
Extended family (>5)	89 (23)
**Residence**	
Hostel	36 (9)
Mess	115 (30)
With family	233 (61)
**Family monthly income, BDT** ^b^	
<20,000	52 (14)
20,000–50,000	216 (56)
>50,000	116 (30)
**BMI status**	
Under weight	57 (15)
Normal weight	206 (54)
Overweight	77 (20)
Obese	44 (11)

*Note:* Descriptive statistics were done.

Abbreviations: BDT, Bangladesh Taka; BMI, body mass index; *M*, mean; SD, standard deviation.

^a^ Religion “Others” included Hindus, Christians, and Buddhists.

^b^ BDT = Bangladesh Taka (currency); $1 = 121.54 BDT as of Feb 2024, 11:08 a.m. UTC.

## 5 Mental Health Outcomes

**FIGURE 2:**
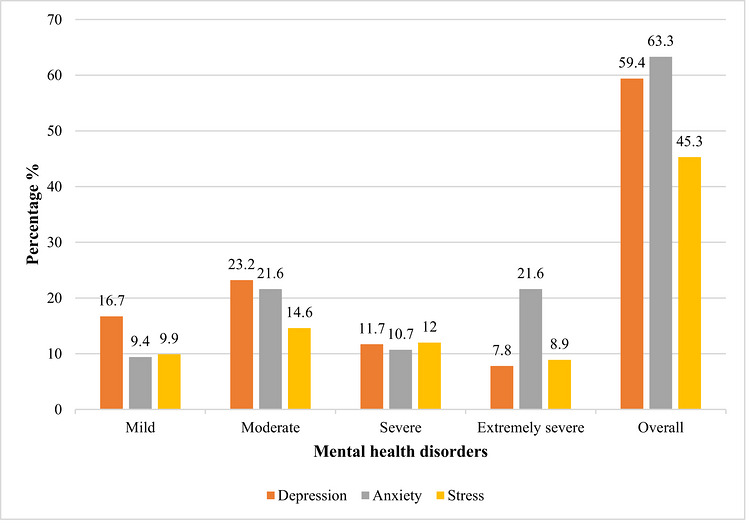
The figure shows the distribution of depression, anxiety, and stress of mental health disorders among the university students in Bangladesh based on the Depression Anxiety and Stress Scale (DASS‐21) scores (*N* = 384).

Depression, anxiety, and stress were assessed using the DASS‐21 scale. As shown in Figure 2, 23.2% of participants reported moderate depression, whereas 7.8% reported extremely severe depression. For anxiety, 21.6% reported mild to extremely severe symptoms, and 14.6% reported mild stress, with 8.9% experiencing extremely severe stress.

Table 2 presents the prevalence of depression, anxiety, and stress across demographic and behavioral patterns.

**TABLE 2:** The association between eating behaviors and mental health problems among the study participants (*N* = 384) using the bivariate chi‐square test.

	Depression			Anxiety			Stress		
	Yes	No		Yes	No		Yes	No
Variables	*n* (%)		*p* value	*n* (%)		*p* value	*n* (%)		*p* value
**Age**			0.6			0.3			0.7
18–22	139 (58)	99 (42)		146 (61)	92 (39)		180 (76)	58 (24)	
23–27	89 (61)	57 (39)		97 (66)	49 (34)		108 (74)	38 (26)	
**Sex**			0.091			**<0.001**			0.067
Male	116 (56)	93 (44)		116 (56)	93 (44)		149 (71)	60 (29)	
Female	112 (64)	63 (36)		127 (73)	48 (27)		139 (79)	36 (21)	
**Educational status**			0.3			**0.029**			0.8
Undergraduate	216 (59)	151 (41)		228 (62)	139 (38)		276 (75)	91 (25)	
Postgraduate	12 (71)	5 (29)		15 (88)	2 (12)		12 (71)	5 (29)	
**Family size, members**			0.5			0.9			0.8
Nuclear family (≤5)	178 (60)	117 (40)		186 (63)	109 (37)		222 (75)	73 (25)	
Extended family (>5)	50 (56)	39 (44)		57 (64)	32 (36)		66 (74)	23 (26)	
**Residence**			0.8			0.5			0.14
With family	135 (58)	98 (42)		144 (62)	89 (38)		168 (72)	65 (28)	
Hostel	22 (61)	14 (39)		26 (72)	10 (28)		26 (72)	10 (28)	
Mess	71 (62)	44 (38)		73 (63)	42 (37)		94 (82)	21 (18)	
**Religion**			0.5			0.5			0.3
Islam	202 (60)	135 (40)		211 (63)	126 (37)		250 (74)	87 (26)	
Others	26 (55)	21 (45)		32 (68)	15 (32)		38 (81)	9 (19)	
**Family monthly income, BDT**			0.6			0.3			0.2
<20,000	33 (63)	19 (37)		28 (54)	24 (46)		34 (65)	18 (35)	
20,000–50,000	124 (57)	92 (43)		139 (64)	77 (36)		164 (76)	52 (24)	
>50,000	71 (61)	45 (39)		76 (66)	40 (34)		90 (78)	26 (22)	
**BMI status**			0.9			0.7			0.5
Under weight	35 (61)	22 (39)		40 (70)	17 (30)		42 (74)	15 (26)	
Normal weight	124 (60)	82 (40)		128 (62)	78 (38)		154 (75)	52 (25)	
Overweight	44 (57)	33 (43)		49 (64)	28 (36)		62 (81)	15 (19)	
Obese	25 (57)	19 (43)		26 (59)	18 (41)		30 (68)	14 (32)	
**Do you take breakfast?**			0.094			0.059			0.052
Always	64 (58)	46 (42)		69 (63)	41 (37)		81 (74)	29 (26)	
Regularly	52 (51)	49 (49)		55 (54)	46 (46)		68 (67)	33 (33)	
Irregularly	112 (65)	61 (35)		119 (69)	54 (31)		139 (80)	34 (20)	
**Do you take lunch?**			**0.018**			**0.007**			0.093
Always	81 (62)	49 (38)		85 (65)	45 (35)		100 (77)	30 (23)	
Irregularly	61 (69)	27 (31)		66 (75)	22 (25)		72 (82)	16 (18)	
Regularly	86 (52)	80 (48)		92 (55)	74 (45)		116 (70)	50 (30)	
**Do you take dinner?**			0.2			0.2			0.2
Always	73 (59)	51 (41)		81 (65)	43 (35)		88 (71)	36 (29)	
Irregularly	56 (67)	27 (33)		58 (70)	25 (30)		68 (82)	15 (18)	
Regularly	99 (56)	78 (44)		104 (59)	73 (41)		132 (75)	45 (25)	
**How often do you eat outside?**			0.7			0.8			0.8
Always	46 (64)	26 (36)		47 (65)	25 (35)		56 (78)	16 (22)	
Irregularly	131 (58)	94 (42)		139 (62)	86 (38)		167 (74)	58 (26)	
Regularly	51 (59)	36 (41)		57 (66)	30 (34)		65 (75)	22 (25)	
**What type of food do you prefer most?**			**0.001**			**0.011**			**0.021**
Rice and meat	127 (62)	79 (38)		136 (66)	70 (34)		155 (75)	51 (25)	
Rice and fish	37 (43)	50 (57)		43 (49)	44 (51)		56 (64)	31 (36)	
Vegetables	40 (68)	19 (32)		39 (66)	20 (34)		50 (85)	9 (15)	
Others^a^	24 (75)	8 (25)		25 (78)	7 (22)		27 (84)	5 (16)	
**Do you take snacks?**			0.6			**0.028**			0.7
Yes	181 (60)	120 (40)		199 (66)	102 (34)		227 (75)	74 (25)	
No	47 (57)	36 (43)		44 (53)	39 (47)		61 (73)	22 (27)	
**Do you prefer junk food?**			0.9			0.8			>0.9
Yes	140 (60)	95 (40)		150 (64)	85 (36)		176 (75)	59 (25)	
No	88 (59)	61 (41)		93 (62)	56 (38)		112 (75)	37 (25)	
**Do you prefer soft drinks?**			0.5			0.7			0.8
Yes	171 (58)	122 (42)		184 (63)	109 (37)		219 (75)	74 (25)	
No	57 (63)	34 (37)		59 (65)	32 (35)		69 (76)	22 (24)	
**The pattern of meals was mostly skipped in a day**			0.9			0.3			0.6
Breakfast	148 (59)	101 (41)		150 (60)	99 (40)		182 (73)	67 (27)	
Lunch	41 (56)	32 (44)		51 (70)	22 (30)		56 (77)	17 (23)	
Dinner	35 (64)	20 (36)		38 (69)	17 (31)		45 (82)	10 (18)	
Never	4 (57)	3 (43)		4 (57)	3 (43)		5 (71)	2 (29)	
**How long do you sleep/day?**			0.6			0.7			0.4
<7 h	96 (61)	61 (39)		101 (64)	56 (36)		114 (73)	43 (27)	
>7 h	132 (58)	95 (42)		142 (63)	85 (37)		174 (77)	53 (23)	
**Please state your smoking history**			0.2			0.5			0.6
Current smoker	55 (68)	26 (32)		56 (69)	25 (31)		64 (79)	17 (21)	
Ex‐smoker	24 (60)	16 (40)		25 (63)	15 (38)		29 (73)	11 (28)	
Never smoker	149 (57)	114 (43)		162 (62)	101 (38)		195 (74)	68 (26)	
**Do you drink alcohol?**			0.2			0.2			0.089
Yes	29 (69)	13 (31)		30 (71)	12 (29)		36 (86)	6 (14)	
No	199 (58)	143 (42)		213 (62)	129 (38)		252 (74)	90 (26)	
**Do you currently exercise?**			0.6			0.12			0.6
Regular	32 (53)	28 (47)		33 (55)	27 (45)		42 (70)	18 (30)	
Irregularly	78 (60)	52 (40)		78 (60)	52 (40)		100 (77)	30 (23)	
No practice	118 (61)	76 (39)		132 (68)	62 (32)		146 (75)	48 (25)	
**Fish (days/week)**			0.4			0.5			0.7
<3	128 (58)	94 (42)		144 (65)	78 (35)		168 (76)	54 (24)	
>3	100 (62)	62 (38)		99 (61)	63 (39)		120 (74)	42 (26)	
**Meat (days/week)**			0.7			0.14			0.6
<3	125 (60)	82 (40)		138 (67)	69 (33)		153 (74)	54 (26)	
>3	103 (58)	74 (42)		105 (59)	72 (41)		135 (76)	42 (24)	
**Egg (days/week)**			0.9			0.9			0.066
<3	126 (59)	87 (41)		135 (63)	78 (37)		152 (71)	61 (29)	
>3	102 (60)	69 (40)		108 (63)	63 (37)		136 (80)	35 (20)	
**General fruits (days/week)**			0.6			0.6			0.5
<3	117 (58)	84 (42)		130 (65)	71 (35)		148 (74)	53 (26)	
>3	111 (61)	72 (39)		113 (62)	70 (38)		140 (77)	43 (23)	
**Ghee (days/week)**			0.7			0.8			0.2
<3	170 (59)	119 (41)		182 (63)	107 (37)		212 (73)	77 (27)	
>3	58 (61)	37 (39)		61 (64)	34 (36)		76 (80)	19 (20)	
**Lentils (days/week)**			0.8			0.2			0.8
<3	163 (59)	113 (41)		180 (65)	96 (35)		208 (75)	68 (25)	
>3	65 (60)	43 (40)		63 (58)	45 (42)		80 (74)	28 (26)	
**Shingara** ^b^ **(days/week)**			0.6			**0.030**			0.8
<3	124 (58)	89 (42)		145 (68)	68 (32)		161 (76)	52 (24)	
>3	104 (61)	67 (39)		98 (57)	73 (43)		127 (74)	44 (26)	
**Pur**i^c^ **(days/week)**			0.6			0.076			0.6
<3	131 (61)	85 (39)		145 (67)	71 (33)		164 (76)	52 (24)	
>3	97 (58)	71 (42)		98 (58)	70 (42)		124 (74)	44 (26)	
**Tea/coffee (days/week)**			0.5			0.7			0.7
<3	135 (58)	98 (42)		149 (64)	84 (36)		173 (74)	60 (26)	
>3	93 (62)	58 (38)		94 (62)	57 (38)		115 (76)	36 (24)	
**Vegetables (days/week)**			0.9			0.4			0.2
<3	125 (59)	87 (41)		138 (65)	74 (35)		164 (77)	48 (23)	
>3	103 (60)	69 (40)		105 (61)	67 (39)		124 (72)	48 (28)	

*Note:* A bivariate chi‐square test was performed; bold values indicated significant findings; *p *≤ 0.05 was considered the level of significance.

Abbreviations: BDT, Bangladesh Taka; BMI, body mass index.

^a^ Other included fast food, sweets, and processed items.

^b^ Singara is a traditional Bangladeshi deep‐fried snack, typically filled with spiced potatoes or minced meat; considered energy‐dense and low in nutritional value.

^c^ Puri is a local deep‐fried flatbread commonly consumed with curry; often categorized as an oil‐rich, refined carbohydrate‐based snack.

### 5.1 Demographic Patterns

Older students reported higher rates of depression (61%), anxiety (66%), and stress (74%). Female students had higher rates of anxiety (73%) than males. Postgraduate students had higher rates of anxiety (88%) than undergraduates. Students living with their families reported lower rates of depression (58%) and anxiety (62%) than those living in hostels.

### 5.2 Body Weight Patterns

Underweight students had higher rates of depression (61%) and anxiety (70%), whereas overweight students had higher stress levels (81%).

### 5.3 Eating Behaviors

Students who consumed breakfast, lunch, or dinner irregularly reported higher rates of depression, anxiety, and stress (65%–82%). Preference for fast food, sweets, and processed items was associated with higher rates of mental health problems, whereas preference for rice, meat/fish, or vegetables was associated with lower rates. Snack consumption was associated with higher anxiety (66%).

### 5.4 Substance Use and Lifestyle

Students who did not smoke or drink alcohol had lower rates of depression, anxiety, and stress. Regular physical activity was associated with lower rates of all mental health outcomes.

Table 3 presents the results of the multivariate logistic regression analysis. The multivariate analysis showed that female students were less likely to report anxiety and stress, and undergraduates were less likely to report anxiety than postgraduates. Regular breakfast and lunch consumption was associated with lower odds of depression, anxiety, and stress, whereas irregular meals were linked to higher odds. Preference for traditional foods, such as rice and fish, was associated with lower odds of depression, whereas alcohol consumption was associated with higher odds of stress. These findings highlight behavioral associations with mental health outcomes, but causality cannot be inferred due to the cross‐sectional design. The sensitivity analyses showed results consistent with the main findings.

**TABLE 3:** Multivariable logistic regression model showing the factors associated with mental health problems among the study participants (*N* = 384).

	Depression			Anxiety			Stress		
Variables	OR	95% CI	*p* value	OR	95% CI	*p* value	OR	95% CI	*p* value
**Age**									
18–22	Ref								
23–27	1.25	0.79, 1.99	0.4	1.32	0.80, 2.17	0.3	0.79	0.47, 1.35	0.4
**Sex**									
Male	0.63	0.39, 1.01	0.054	0.41	0.25, 0.69	**<0.001**	0.48	0.27, 0.83	**0.010**
Female	Ref								
**Educational status**									
Undergraduate	0.51	0.15, 1.52	0.2	0.17	0.03, 0.67	**0.026**	1.21	0.35, 3.68	0.7
Postgraduate	Ref								
**Family size, members**									
Nuclear family (≤5)	1.09	0.65, 1.81	0.8	0.76	0.44, 1.31	0.3			
Extended family (>5)	Ref								
**Residence**									
With family	0.90	0.40, 1.95	0.8	0.52	0.21, 1.23	0.15	1.09	0.44, 2.57	0.8
Mess	1.42	0.60, 3.29	0.4	0.86	0.33, 2.14	0.7	2.56	0.96, 6.66	0.055
Hostel	Ref								
**Religion**									
Islam	Ref								
Others	0.65	0.33, 1.29	0.2	1.07	0.51, 2.33	0.9	1.36	0.60, 3.33	0.5
**Family monthly income, BDT**									
<20,000	Ref								
20,000–50,000	0.60	0.30, 1.18	0.15	1.36	0.67, 2.74	0.4	1.66	0.80, 3.42	0.2
>50,000	0.65	0.30, 1.36	0.3	1.31	0.60, 2.83	0.5	1.81	0.80, 4.07	0.15
**BMI status**									
Normal weight	Ref								
Obese	0.72	0.30, 1.73	0.5	0.50	0.19, 1.27	0.14	0.63	0.24, 1.64	0.3
Overweight	0.62	0.28, 1.35	0.2	0.61	0.26, 1.42	0.3	1.20	0.48, 2.99	0.7
Under weight	0.85	0.44, 1.64	0.6	0.70	0.33, 1.43	0.3	0.95	0.45, 1.96	0.9
**Do you take breakfast?**									
Always	0.68	0.35, 1.31	0.3	0.62	0.31, 1.25	0.2	0.59	0.27, 1.26	0.2
Regularly	0.64	0.37, 1.10	0.11	0.59	0.33, 1.05	0.072	0.52	0.28, 0.98	**0.042**
Irregularly	Ref								
**Do you take lunch?**									
Always	0.88	0.43, 1.79	0.7	0.85	0.39, 1.84	0.7	0.93	0.40, 2.14	0.9
Regularly	0.50	0.27, 0.89	**0.020**	0.44	0.23, 0.84	**0.013**	0.55	0.27, 1.08	0.088
Irregularly	Ref								
**What type of food do you prefer most?**									
Rice and meat	0.59	0.23, 1.40	0.2	0.75	0.27, 1.91	0.6	0.84	0.26, 2.30	0.8
Rice and fish	0.26	0.09, 0.66	**0.006**	0.36	0.12, 0.97	0.050	0.53	0.16, 1.54	0.3
Vegetables	0.73	0.26, 1.97	0.5	0.64	0.21, 1.85	0.4	1.28	0.34, 4.43	0.7
Others	Ref								
**Do you take snacks?**									
Yes				1.35	0.75, 2.41	0.3			
No				Ref					
**Do you currently exercise**?									
Regular				0.91	0.45, 1.85	0.8			
No practice				1.37	0.82, 2.31	0.2			
Irregularly				Ref					
**Meat (days/week)**									
<3				Ref					
>3				0.87	0.54, 1.40	0.6			
**Shingara (days/week)**									
<3				Ref					
>3				0.67	0.39, 1.13	0.13			
**Puri (days/week)**									
<3				Ref					
>3				0.79	0.47, 1.35	0.4			
**Egg (days/week)**									
<3							Ref		
>3							1.61	0.96, 2.72	0.074
**Do you drink alcohol?**									
No							Ref		
Yes							2.65	1.08, 7.59	**0.048**

*Note:* Multivariable logistic regression was done. Bold values indicated significant findings; *p* ≤ 0.05 was considered the level of significance. CI and OR imply confidence interval and odds ratio.

Abbreviations: BDT, Bangladesh Taka; BMI, body mass index; OR, odds ratio.

## 6 Discussions

Over the past few decades, alterations in diet and lifestyle have been linked to rising rates of malnutrition and chronic diseases related to diet [32]. University students, navigating the shift to independent living, are susceptible to unhealthy eating patterns [33], which, as our findings indicate, are significantly associated with higher levels of stress, anxiety, and depression among private university students. Conversely, regular meals and healthier dietary choices, such as traditional foods, appeared to protect mental well‐being. Taken together, these results underscore the importance of integrating nutrition education and mental health support into university programs to promote overall student well‐being and resilience.

The study found that 15% of participants were underweight, 20% overweight, and 11% obese, indicating that nearly half (46%) of the sample had some form of weight‐related concern. Compared to a previous study of Bangladeshi university students [23], the current sample had a higher proportion of underweight individuals (15% vs. 9.5%) and a markedly lower rate of obesity (11% vs. 21.7%), suggesting a shift in weight distribution within this population. This shift may reflect changing dietary patterns, increased physical activity, or greater health awareness among university students in recent years. Urbanization, cultural factors, and evolving food environments could also contribute to these differences.

The current study found elevated prevalence rates of mental health problems among participants, with depression at 59.4%, anxiety at 63.3%, and stress at 45.4%. Compared with a previous study of Bangladesh university students [23], the prevalence in the present study was substantially higher for depression (59.4% vs. 33.20%) and anxiety (63.3% vs. 29.1%). However, the prevalence of stress was lower than that reported in another study conducted among public students (45.4% vs. 62.5%) [34]. It is worth noting that the earlier study [23] primarily included students from public universities, which may partly explain the observed differences. Variations in academic environment, lifestyle patterns, and psychological stressors between public and private universities may also contribute to these findings [9]. These findings highlight the need for strengthened university‐based mental health support and lifestyle‐focused interventions for students.

Mental health problems were observed among both male and female students; however, male students were less likely to report anxiety and stress than females, which is consistent with previous studies [24, 35, 36, 37]. Female students may experience higher levels of psychological distress due to a combination of academic, socioeconomic, and cultural factors [38, 39, 40]. At the same time, some studies have reported that male students can also experience substantial psychological distress [23, 29, 41, 42, 43], potentially related to academic pressures and societal norms that discourage emotional expression [43, 44, 45]. These findings suggest the need for university‐based mental health support programs that address gender‐related vulnerabilities.

This study further found that undergraduate students were less likely to report anxiety compared with postgraduate students. This difference may reflect the cumulative pressures that emerge as students progress through university [46]. In Bangladesh's highly competitive job market, where unemployment rates remain high, students approaching graduation may experience greater concerns about future career prospects [47]. Combined with demanding academic requirements and a competitive university environment, these pressures may be associated with higher levels of depression, anxiety, and stress among students in the later years of study [15, 48]. These findings highlight the importance of strengthening mental health support services for students in the later stages of university education.

This study found that students who regularly consumed both breakfast and lunch were less likely to report symptoms of stress, anxiety, and depression compared with those with irregular meal patterns. Similar associations have been reported in a systematic review and meta‐analysis [49], a multicountry study among university students [50], and research conducted in Spain [51]. Previous studies also suggest that meal skipping and other unhealthy eating behaviors are associated with poorer mental health outcomes, potentially through stress‐related hormonal responses, such as cortisol and activation of the hypothalamic–pituitary–adrenal (HPA) axis [52, 53, 54, 55, 56]. These findings highlight the importance of promoting regular meal patterns through university‐based health promotion and nutrition awareness programs.

The study also found that students who preferred traditional foods, such as rice and fish, were less likely to report depression compared to those who preferred fast food, sweets, and processed items. This finding is consistent with previous research linking unhealthy dietary patterns to poorer mental health outcomes [19, 56, 57]. Globally, the consumption of ultra‐processed foods, fast food, and unhealthy snacks is increasing [58], and such foods are typically high in energy, fat, sugar, and salt while being low in micronutrients and fiber [59]. These dietary patterns have been associated with inflammation and oxidative stress, which may influence mental well‐being [60]. These dietary patterns have been associated with inflammation and oxidative stress, which may influence mental well‐being.

The study found that students who consumed alcohol were more likely to report stress compared with nondrinkers, consistent with previous research [61, 62, 63, 64]. Alcohol consumption is associated with poorer mental health outcomes through both biological and psychosocial pathways [65, 66, 67, 68, 69, 70, 71, 72, 73, 74, 75, 76]. Excessive alcohol use may increase stress in academic and home environments. These findings highlight the need for university‐based awareness programs and interventions addressing alcohol use, alongside promoting healthy lifestyle behaviors, including regular meals, to support student mental well‐being.

### 6.1 Strengths and Limitations

A notable strength of this study was the use of directly measured height and weight rather than relying on self‐reported values. Direct measurements minimize the risk of reporting bias and improve the accuracy of anthropometric data, thereby enhancing the reliability of the findings [60]. By including students from multiple universities, the study strengthens its geographical representation. Validated tool, DASS‐21 for depression, anxiety, and stress ensured robust, reliable, and comparable results.

However, our study had limitations. First, the 19‐item questionnaire assessed dietary behaviors, such as meal frequency, food choices, junk food, and alcohol consumption, rather than detailed nutrient intake, and was not validated against standard tools, such as FFQ or 24‐h recall. Consequently, the findings should be interpreted as exploratory and descriptive rather than definitive measures of dietary intake. The use of convenience sampling may further limit generalizability. We invited students from all levels of study to participate in our survey. The composition of educational status, 96% undergraduate and only 4% postgraduate students, does not reflect the demographics of the university students’ level of study. One reason for the low number of postgraduate students is that they were less interested in participating due to time constraints and an overloaded curriculum. Third, convenience sampling was used, which may limit the generalizability of the results to all private university students in Dhaka North City Corporation. Finally, although collapsing the original three frequency categories (<3, 3, and >3 days/week) into two (<3 vs. >3 days/week) improved statistical stability and interpretability, this adjustment may limit direct comparability with studies using the original categorization.

## 7 Conclusions

This study found a significant association between unhealthy eating behaviors and mental health problems among university students, with meal skipping, fast food, sweets, processed foods, junk food, and alcohol consumption emerging as positive predictors of depression, anxiety, and stress. These findings highlight the interconnected nature of dietary habits and psychological well‐being in this population.

Practically, the results support the need for integrated university‐based interventions that combine nutrition education, mental health screening, and counseling services. Multidisciplinary support models involving dietitians and mental health professionals may help address both dietary risks and psychological distress simultaneously.

Given the cross‐sectional design, causal relationships cannot be established. Future longitudinal and intervention studies are needed to clarify directionality and evaluate the effectiveness of integrated nutrition–mental health programs in university settings.

## Author Contributions

Conceptualization: Mst. Mahfuza Akter and Taslima Khatun. Validation: Mst. Mahfuza Akter, Taslima Khatun, and Sheikh Mohammed Shariful Islam. Supervision: Taslima Khatun and Sheikh Mohammed Shariful Islam. Data curation and formal analysis: Mst. Mahfuza Akter and Md Nazmus Sakib. Original draft writing: Mst. Mahfuza Akter. Methodology, project administration and visualization: Mst. Mahfuza Akter, Taslima Khatun, and Sheikh Mohammed Shariful Islam. Resource: Mst. Mahfuza Akter, Taslima Khatun, Md Nazmus Sakib, and Sheikh Mohammed Shariful Islam. Software: Mst. Mahfuza Akter and Md Nazmus Sakib. Writing – review and editing: all authors.

## Funding

The authors have nothing to report.

## Ethics Statement

This study was approved by the ethical research committee of the Bangladesh University of Health Sciences (Memo No: BUHS/ERC/EA/23/394). All methods were performed in accordance with the relevant guidelines and regulations, specifically the ethical principles outlined in the Declaration of Helsinki for research involving human participants.

## Consent

Informed consent was obtained from all subjects before inclusion in the study.

## Conflicts of Interest

The authors declare no conflicts of interest.

## Supporting information



puh270283‐sup‐0001‐SuppMat1.docx

puh270283‐sup‐0002‐SuppMat2.docx

## Data Availability

The data that support the findings of this study are openly available in Mendeley Data Repository (https://data.mendeley.com/datasets/79ng9n3mfz/1) and can be used for academic purposes upon request to the corresponding author.
