# Supplementary material for: Dietary Behaviors and Mental Health Disorders Among Private University Students in Dhaka North City Corporation, Bangladesh: A Cross‐Sectional Study
Source: Public Health Chall. 2026 May 28;5(2):e70283. doi: 10.1002/puh2.70283 (PMC13239289; doi:10.1002/puh2.70283)
Supplement: Supplementary file 1 — puh270283‐sup‐0001‐SuppMat1.docx [file PUH2-5-e70283-s002.docx]

**Supplementary File 1. The Adapted 19-Item Questionnaire**

| **SL** | **Questions** | **Responses** |
| --- | --- | --- |
|  | Do you take breakfast? | - Always - Regularly - Irregularly |
|  | Do you take lunch? | - Always - Regularly - Irregularly |
|  | Do you take dinner? | - Always - Regularly - Irregularly |
|  | How often do you eat outside? | - Always - Regularly - Irregularly |
|  | What type of food do you prefer most? | - Rice and meat - Rice and fish - Vegetables - Others (fast food, sweets, and processed items) |
|  | Do you take snacks? | - Yes - No |
|  | Do you prefer junk food? | - Yes - No |
|  | Do you prefer soft drinks? | - Yes - No |
|  | The pattern of meals was mostly skipped in a day. | - Breakfast - Lunch - Dinner - Never |
| **Please indicate how many days per week you usually eat the following foods. Based on your answer, tick the most appropriate option.** | | |
|  | Fish (days/week) | - *<3* - *>3* |
|  | Meat (days/week) | - *<3* - *>3* |
|  | Egg (days/week) | - *<3* - *>3* |
|  | General fruits (days/week) | - *<3* - *>3* |
|  | Vegetables (days/week) | - *<3* - *>3* |
|  | Ghee (days/week) | - *<3* - *>3* |
|  | Lentils (days/week) | - *<3* - *>3* |
|  | Shingara (days/week) | - *<3* - *>3* |
|  | Puri (days/week) | - *<3* - *>3* |
|  | Tea/coffee (days/week) | - *<3* - *>3* |
